# A quantitative dissection of the DNA‐binding properties of pathogenic GATA1 mutants

**DOI:** 10.1111/febs.70614

**Published:** 2026-06-01

**Authors:** Kaoru Takasaki

**Affiliations:** ^1^ Division of Hematology Children's Hospital of Philadelphia Philadelphia PA USA; ^2^ Department of Pediatrics Perelman School of Medicine at the University of Pennsylvania Philadelphia PA USA

**Keywords:** DNA–protein binding, erythropoiesis, GATA1, GATA1s, native holdup assay

## Abstract

Transcription factors are modulated by a precisely coordinated set of conditions, including cell context, target sequences and their accessibility, and co‐factor recruitment. Disruption to any of these conditions can dramatically affect transcription factor activity, but quantitatively characterizing the consequences of individual mutations—either in the transcription factors themselves or in their target sequences—has remained a technical challenge. Zambo *et al.* present an innovation on their native holdup assay that measures DNA–protein binding activity under physiologic or near‐physiologic conditions and use mutant GATA1‐*ATP2B4* binding as an illustrative example. This technique holds promise for uncovering the molecular mechanisms underlying genetically‐driven diseases.

## Graphical abstract

Transcription factor activity is influenced by cell context, accessibility of target sequences, and co‐factor recruitment. Quantitatively characterizing the consequences of individual mutations in the transcription factors or their target sequences remains technically challenging. Zambo *et al.* use mutant GATA1‐ATP2B4 binding to illustrate an innovation on their native holdup assay that measures DNA–protein binding activity under physiologic or near‐physiologic conditions. This technique holds promise for uncovering the molecular mechanisms underlying genetically‐driven diseases.

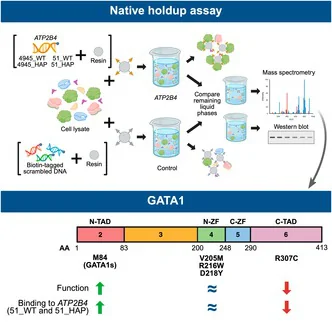

**Abbreviations**
ATP2B4: ATPase plasma membrane Ca^2+^ transporting 4
C‐TAD: Carboxy‐terminus transactivation domain
C‐ZF: Carboxy zinc finger domain
EMSA: Electrophoretic mobility shift assay
FOG1: Friend of GATA1
GATA1: GATA binding protein 1
ML‐DS: Myeloid leukemia of Down syndrome
nHU: Native holdup
N‐TAD: Amino‐terminus transactivation domain
N‐ZF: Amino zinc finger domain
PBM: Protein binding microarray
TAL1: T‐cell acute lymphoblastic leukemia protein 1
TAM: Transient abnormal myelopoiesis
WT: Wild-type

## Introduction

**Fig. 1:**
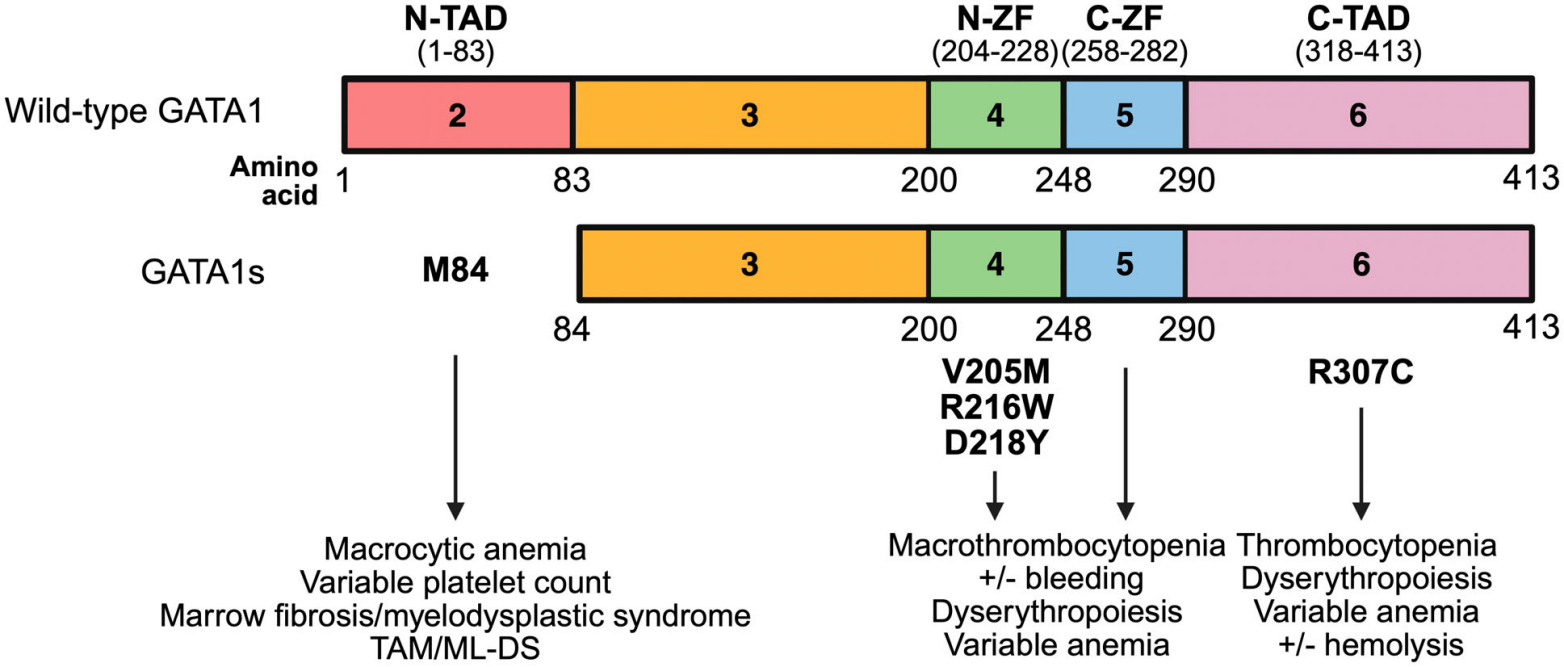
Illustration of human GATA1 (wild‐type and GATA1s isoforms). Specific mutations investigated by Zambo *et al*. are highlighted, along with broad genotype–phenotype associations.

GATA binding protein 1 (GATA1) is a hematopoietic transcription factor critical for normal erythropoiesis and megakaryopoiesis [1]. The human protein consists of four functional domains: the amino and carboxy transactivation domains (N‐TAD and C‐TAD, respectively) and the amino and carboxy zinc fingers (N‐ZF and C‐ZF, respectively) (Fig. 1). The TADs have both specific and redundant functions in regulating their target genes, and both are considered necessary for normal erythropoiesis and megakaryopoiesis. The N‐ZF stabilizes GATA1 binding to its DNA targets and to its key co‐factor Friend of GATA1 (FOG1), while the C‐ZF is essential for binding to the single, palindromic, and complex inverted/repeated double tandem GATA1 target motifs.

Mutations in GATA1 have been implicated in variable cytopenias as well as transient abnormal myelopoiesis (TAM) and myeloid leukemia of Down syndrome (ML‐DS), which are seen almost exclusively in infants and children with Down syndrome. Attempts to draw genotype–phenotype correlations have had limited success. In a euploid background, mutations resulting in exclusive expression of GATA1s, the truncated isoform missing the N‐TAD, are associated with dyserythropoietic, normocytic or macrocytic anemias, normal to decreased platelet counts, and occasionally neutropenia. In the context of trisomy 21, GATA1s mutations give rise to TAM, a typically self‐limited myeloproliferative disorder, and subsequently ML‐DS in a subset of cases [2]. Mutations affecting or in close proximity to the zinc fingers (amino acids 200–290) tend to cause thrombocytopenias with decreased platelet function and variable anemias, and similar manifestations have been reported for mutations affecting the C‐TAD. However, there is great clinical variability reported even within these broad correlations, and why mutations in the same functional domain regions or even amino acids give rise to distinct phenotypes remains unresolved.

In this study, Zambo *et al.* [3] present a powerful application of their native holdup (nHU) assay [4] to examine the exact DNA‐binding sequence and protein domain(s) required for a successful interaction, and use GATA1 as an example to illustrate the feasibility and utility of this approach. They quantitatively compare the effects of pathological GATA1 mutations on the binding affinities to the wild‐type (WT) and mutated erythroid‐specific *ATP2B4* promoter. Their innovation offers an *in vitro* method by which to dissect the impact of sequence variants and the function of individual DNA‐binding domains, thus offering a better understanding of the molecular mechanisms underlying genetic diseases.

## The quantitative characterization of transcription factor binding

A key innovation of the nHU assay is its use of endogenous or transfected protein obtained from whole‐cell or nuclear extracts without further purification. The use of cell extracts allows for the measurement of binding affinities between the protein of interest and its DNA targets under near‐physiologic conditions. These cell extracts are incubated with DNA targets, or ‘baits’, and separately with a control; the protein contents of the supernatants can then be compared to identify proteins that are lacking from the experimental condition and thus inferred to interact with the DNA targets. Titration of the DNA ‘bait’ concentration allows the assay to capture subtle differences in DNA–protein interactions that may otherwise have been missed under non‐physiological conditions.

While the Zambo *et al.* here use the erythroid‐specific *ATP2B4* (ATPase plasma membrane Ca^2+^ transporting 4) promoter as the ‘bait’ to assess the DNA‐binding function of mutated GATA1 isoforms, this nHU assay can clearly be applied to identify transcription factors or other proteins that bind to a DNA sequence of interest. Indeed, the authors identified nearly 300 proteins that interacted with their *ATP2B4* baits and were able to determine specificity for binding by GATA1 by comparing the changes in binding affinity between the *ATP2B4* WT and mutated baits. This illustrates the power of the nHU assay to specifically identify novel protein binding partners and the DNA sequence requirements for protein binding.

**Fig. 2:**
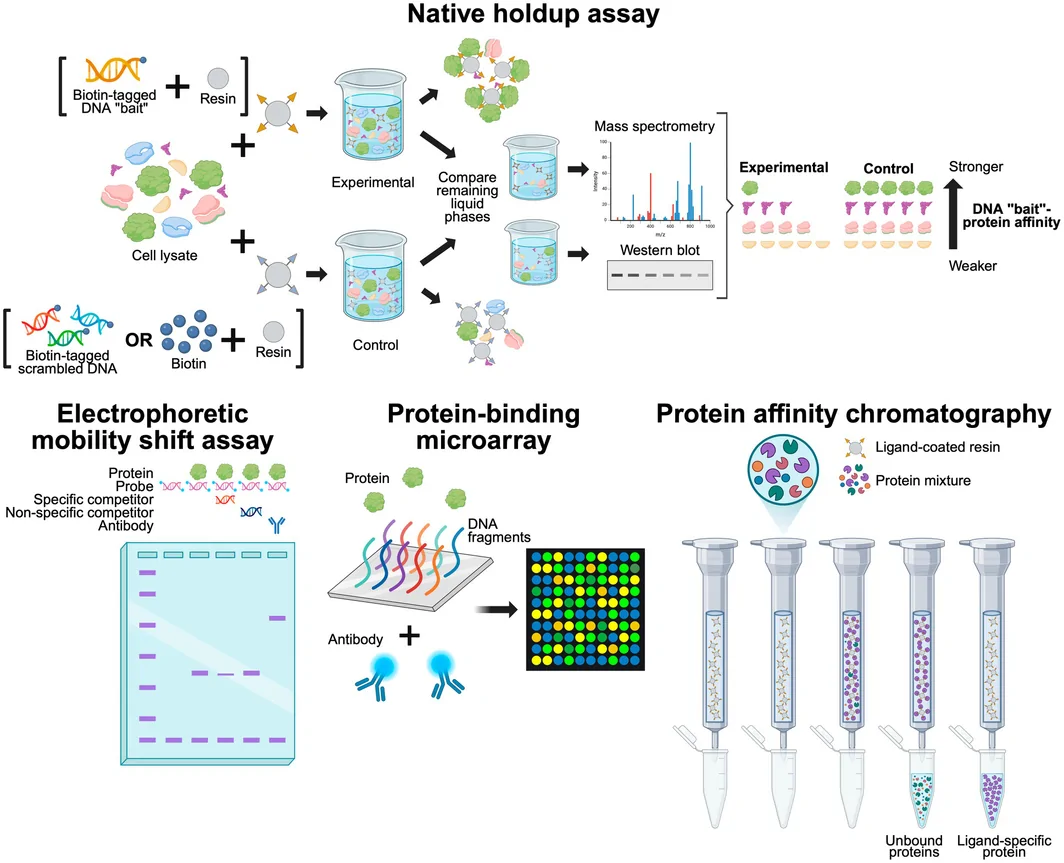
Schematics of native holdup assay, electrophoretic mobility shift assay, protein‐binding microarray, and protein affinity chromatography.

This work builds on decades of efforts to characterize DNA‐binding proteins and their targets (Fig. 2). As the authors point out, the ability to quantify the strength of the binding interaction under physiologic conditions is a significant advantage over existing computational prediction methods and electrophoretic mobility shift assays (EMSAs) [5], the latter which is typically performed under non‐physiologic conditions and may not be sufficiently sensitive to capture subtle changes in binding affinity. In addition, protein‐binding microarrays (PBMs) [6] can identify the target sequence of a transcription factor of interest but cannot identify novel binding proteins of a known sequence and is similarly non‐physiologic and only semi‐quantitative. Protein affinity chromatography [7] can identify target sequence‐binding proteins from an unbiased mixture but relies on non‐physiological conditions.

## The heterogeneity of *in vitro* activity and the clinical consequences of GATA1 mutations

Zambo *et al*. focused on six disease‐associated mutations in GATA1 along with the WT isoform and two artificial mutations disrupting the N‐ and C‐ZFs. They first assessed total protein levels as well as function via an *ATP2B4*‐luciferase reporter assay and found significant discrepancies between GATA1 protein level and function, even between those with similar or proximate mutations. This is reminiscent of the heterogeneity of the clinical phenotype.

V205M [8], R216W [9], and D218Y [10] are well‐known GATA1 mutations affecting the N‐ZF and are associated with varying degrees of anemia and macrothrombocytopenia (Fig. 1). Although they were classified as loss‐of‐function based on the reporter assay, there were only small differences in binding to the *ATP2B4* promoter sequences. This raises future questions about whether the mutations instead affect the binding and recruitment of co‐factors, such as TAL1 (T‐cell acute lymphoblastic leukemia protein 1) [11] and whether this discrepancy between DNA binding and expression by these mutated GATA1 proteins is specific to *ATP2B4* or also occurs with other targets.

In contrast, R307C, which has been associated with hemolytic anemia and thrombocytopenia [12] (Fig. 1), was found to have both decreased function and *ATP2B4* GATA motif binding. This is consistent with prior work suggesting that, although the mutated arginine is outside of the putative boundaries of the C‐ZF, it may play a critical role in DNA binding by extending into the minor groove [13].

Strikingly, the GATA1s isoform was found to have increased activity and DNA binding despite the loss of what is considered a critical transactivation domain. These findings offer potential insight into the overall distinct phenotype reported with GATA1s compared with other mutations, including clinical features of Diamond–Blackfan anemia and progression to myelodysplastic syndrome [1]. The increased binding and activity of GATA1s seen here are distinct from what has been reported by genome‐wide chromatin occupancy studies, in which GATA1s was less effective at binding and inducing erythroid targets [14, 15]. As these studies were performed in the murine G1ME line [14] and a *Gata1s* mouse model [15], species‐specific differences are possible. However, given the anemia seen almost universally in individuals with germline *GATA1s* mutations, it is more likely that the consequences of GATA1 mutations are target‐specific, and additional studies with other GATA1 targets are necessary before broader conclusions about GATA1s binding and activity can be drawn.

## Conclusion

Zambo *et al*. here have developed a sensitive, quantitative method for characterizing how proteins bind to specific DNA sequences and how even point mutations can dramatically affect this interaction. By using known polymorphisms in the erythroid‐specific *ATP2B4* promoter and disease‐associated GATA1 mutations as an illustrative example, they demonstrate the clear clinical applicability of their nHU assay. As the availability and sensitivity of genetic diagnostics continues to expand, techniques such as this will play an important role in determining the significance of detected mutations and the mechanisms underlying genetically‐driven diseases.

## Conflicts of interest

The author declares no conflicts of interest.
